# Molluskicidal nanoemulsion of *Neomitranthes obscura* (DC.) N. Silveira for schistosomiasis control

**DOI:** 10.3389/fphar.2023.1078936

**Published:** 2023-02-24

**Authors:** Leonardo da Silva Rangel, Francisco Paiva Machado, Raquel Amaral, Ana Cláudia Rodrigues Da Silva, Marcelo Guerra Santos, José Augusto Albuquerque Dos Santos, Natalia Lidmar Von Ranke, Carlos Rangel Rodrigues, Leandro Rocha, Robson Xavier Faria

**Affiliations:** ^1^ Laboratório de Avaliação e Promoção da Saúde Ambiental, Instituto Oswaldo Cruz, Rio de Janeiro, RJ, Brazil; ^2^ Programa de Pós Graduação em Ciências e Biotecnologia, Universidade Federal Fluminense, Niterói, RJ, Brazil; ^3^ Laboratório de Tecnologia de Produtos Naturais, Universidade Federal Fluminense, Niterói, RJ, Brazil; ^4^ Programa de Pós Graduação em Biotecnologia Vegetal e Bioprocessos, Universidade Federal do Rio de Janeiro, Rio de Janeiro, RJ, Brazil; ^5^ Departamento de Ciências, Faculdade de Formação de Professores, Universidade do Estado do Rio de Janeiro, Rio de Janeiro, RJ, Brazil; ^6^ Departamento de Ciências Biológicas e da Saúde, Universidade Federal de Amapá, Macapá, AP, Brazil; ^7^ Centro de Formação de Professores, Universidade do Estado do Rio de Janeiro, Rio de Janeiro, RJ, Brazil

**Keywords:** essential oil, *Neomitranthes obscura*, molluskicide, *Biomphalaria*, *Schistosoma mansoni*

## Abstract

Schistosomiasis is caused by the parasite *Schistosoma mansoni*, which uses mollusks of the *Biomphalaria* genus as intermediate hosts. In 2020, approximately 241 million people worldwide underwent treatment for schistosomiasis. For this reason, the World Health Organization encourages research on alternative molluskicides based on plant species. The objective of this work was to investigate *Neomitranthes obscura* essential oil from leaf chemical composition and its essential oil nanoemulsion activity on intermediate hosts of schistosomiasis *Biomphalaria glabrata* control. The major chemical components of the *Neomitranthes obscura* essential oil were zonarene, seline-3,7(11)-diene, β-selinene, and α-selinene. The nanoemulsion tested using 24-well plate methodology showed lethality and juvenile mollusks with LC_90_ values of 53.9 and 25.0 ppm after 48 h, respectively, and on their spawning with an LC_90_ of 66.2 ppm after 48 h. Additionally, the nanoemulsion exhibited an LC_90_ value against the infective form of the parasite *Schistosoma mansoni* of 11.5 ppm after 4 h. This pharmaceutical formulation acted inhibiting the acetylcholinesterase activity and was not toxic for *Mellanoides sp*. This result suggests the use of this nanoformulation as a promising alternative in the control of *Biomphalaria glabrata* and the transmission of schistosomiasis.

## Introduction

*Schistosomiasis* is a parasitic disease caused by the trematode species *Schistosoma mansoni*, which in 2020 affected approximately 240 million people worldwide to undergo treatment for schistosomiasis. *Schistosoma mansoni* requires aquatic intermediate hosts in its infection cycle, such as mollusks from the *Biomphalaria* genus (Neglected_diseases, 2022; Colley et al., 2014).

The World Health Organization (WHO) recommends improving the basic sanitation system and prevention methods, such as eradicating mollusk hosts using chemical pesticides, such as niclosamide, to control the disease (OMS-Organização Mundial da saúde, 1983). However, this substance is toxic to the environment and possesses cases of resistance to this agent, making it necessary to search for new substances (Inobaya et al., 2014). Therefore, the WHO encourages research into alternative molluskicides based on plant derivatives, since these are abundant in countries with endemic schistosomiasis. It is difficult for vegetal species and plant-based products to develop resistance because they are phytocomplex (Tavares et al., 2007; Ding-Feng, 2010).

Although several extracts and vegetable oils have intrinsic molluskicidal activity, they exhibit low solubility in aqueous media due to their hydrophobic characteristics. Nanotechnology has been used to circumvent this problem by promoting the solubility and stability of active substances. Currently, there are several drug nanocarriers, including nanoparticles, nanoemulsions (NEs), and liposomes (Donsì and Ferrar, 2016). Some important advantages of these nanocarriers are their easy preparation, simple composition, low production cost, possibility of industrial production, and high thermodynamic stability (Cornwell et al., 2000; Trommer and Neubert, 2006; Sagalowicz and Leser, 2010; Campos et al., 2012; Jaiswal et al., 2015).

Belonging to the Myrtaceae family, which is one of the families with the highest species richness in Brazilian Restingas, the species *Neomitranthes obscura* is known as “camboim-de-cachorro” and “pitanga-de-cachorro.” The geographic distribution occurs only in Espírito Santo and Rio de Janeiro in Brazil and is popularly used as food and medicine, mainly for the treatment of intestinal disorders. Some biological activities are known, such as *Trypanosoma cruzi* proliferation and cholinesterase activity inhibition (Marques, 2001; Ramos et al., 2010; Faria et al., 2017).

Therefore, we evaluated the *N. obscura* leaf essential oil nanoemulsion properties on *B. glabrata* mollusks and against the human infective form of *S. mansoni* to control the transmission of schistosomiasis.

## Methodology

### Vegetal material

Fresh leaves of *N. obscura* were collected on 27 June 2018, in the Restinga de Jurubatiba National Park, Rio de Janeiro, Brazil (22°13′4.00625° and 41°35.919 W). The collection and research of plant material were authorized by Sisbio/ICMbio (13,659-14) and SisGen (A0D648D). The species was identified by botanist Dr. Marcelo Guerra Santos, and a voucher was deposited in the herbarium of the Faculty of Teacher Training (University of the State of Rio de Janeiro, Brazil).

### Essential oil extraction

Fresh leaves (1,810 g) were separated from the stem and ground in distilled water. Then, the plant material was placed in a 5 L round-bottomed flask and subjected to hydrodistillation for 4 h in a Clevenger-type apparatus. Then, the essential oil was dried with anhydrous sodium sulfate and stored in an amber glass vial at 4°C.

### Essential oil characterization

The oil was characterized using a GC‒MS QP2010 (Shimadzu) gas chromatograph equipped with a mass spectrometer and a GC-2014 (Shimadzu) gas chromatograph equipped with a flame ionization detector (FID). Gas chromatographic (GC) conditions were as follows: injector temperature, 260°C; ca, helium as carrier gas; flow rate, 1 mL/min and split injection with split ratio 1:40. The oven temperature was initially 60°C and then increased to 290°C at a 3°C/min rate. One microliter of the sample dissolved in dichloromethane (1:100 mg/μL) was injected into an RTX-5 column (0.25 mm ID, 30 m in length, 0.25 μm, and film thickness). Mass spectrometry (MS) electron ionization was 70 eV, and the scan rate was one scan/s. The ionization gas chromatography GC-FID conditions were similar to those of the MS, except for the FID temperature at 290°C. The arithmetic index (AI) was calculated by interpolating the retention times of a mixture of aliphatic hydrocarbons (C9–C30) analyzed under the same conditions. The identification of substances was accomplished by comparing their retention indices and mass spectra with those reported in the literature (Adams, 2007). The MS fragmentation pattern of compounds was also compared with NIST mass spectrum libraries. The relative abundance of the chemical constituents was determined by flame GC-FID under the same conditions as GC‒MS. The FID peak area normalization method obtained the analysis and percentages of these compounds.

### Nanoemulsion preparation and characterization

Emulsification was performed by the low-energy input method by phase inversion in temperature (PIT) according to Feng et al. (2021). The oil-in-water (O/W) emulsions were composed of 5% (w/w) *N. obscura* essential oil, 5% (w/w) surfactant mixture, and 90% (w/w) water. Surfactants (Span 80 and Tween 80) with an hydrophilic-lipophilic balance (HLB) range of 12–15 were used. For the preparation of NEs, the oily phase consisted of the essential oil of *N. obscura,* and a mixture of surfactants was homogenized by magnetic stirring (500 rpm) for 30 min. In sequence, the oil phase was heated to 40°C, the aqueous phase (distilled water) was heated to 40°C ± 2°C, and then the aqueous phase was slowly dripped onto the oil phase under constant magnetic stirring for another 60 min. The formulations were characterized by dynamic light scattering (DLS) in a Zetasizer (Malvern, United Kingdom). The NEs were diluted in distilled water (1:40), and the parameters analyzed were droplet size (nm) and polydispersity index (PdI). Additionally, the turbidity of the formulations was evaluated in a UV‒visible spectrophotometer (T80 UV/VIS Spectrometer, PG Instruments Ltd.) at a wavelength of 570 nm using a quartz cuvette with an optical path of 1 cm and distilled water as a blank.

### Molluskicidal assay

The bioassay was performed with *B. glabrata* mollusks by the method of Santos et al. (2017). For the test, the animals were separated into groups of three individuals for each evaluated developmental stage. Adult and juvenile snails with diameters of 10–12 mm and 6–8 mm, respectively, were individually placed in 24-well plates and exposed to NE at concentrations of 20–120 ppm with a 2 mL final volume. The mortality was compared with NE blank and distilled water and with niclosamide as a pharmacological control at 2 mg/L. Then, mortality was assessed at 24 and 48 h. The absence of retraction in the shell and hemolymph release were the criteria for assessing mortality. The test was performed in triplicate, on different days.

### Ovicidal assay

Styrofoam plates were deposited in the water of the *B. glabrata* breeding tanks for oviposition After 48 h, the egg capsules were carefully removed from the Styrofoam and placed in 24-well plates using the adapted method of Araújo et al. (2019). Then, the viable eggs were counted at time zero, and 1 mL of NE was added to the wells at concentrations of 20–120 ppm. After 24 and 48 h of exposure, viable egg counts were repeated.

### Cercaricidal assay

In 24-well plates, the amount of *S. mansoni* cercariae present in 1 mL was initially estimated using 20 μL of Lugol’s and counted under a stereomicroscope, where we obtain an average of 80 cercariae per plate well for these assays. Then, in another well of the plate, 1 mL of the suspension of *S. mansoni* cercariae and 1 mL of NE were added at concentrations of 20–120 ppm. Then, 20 μL of 0.1% Trypan Blue dye was added. The counting of dead cercariae, stained blue, was performed from 1 to 4 h.

For tests with mollusks of another genus, snails *Mellanoides* sp. with 15–20 mm in diameter (length), collected from the IOC/FIOCRUZ ditches, kept in dechlorinated water and fed with lettuce leaves for stabilization, at the Pavilion Lauro Travassos, at Instituto Oswaldo Cruz, in the state of Rio of January. We used the methodology adapted with 24-well plates, previously described for *B. glabrata* (Santos et al., 2017). The substances were tested at lethal concentrations for 50% and 90% (LC_50_ and LC_90_) of that obtained for *B. glabrata*. After the treatment for 48 h, the mollusks were recovered for 96 h in H_2_O. The mortality was compared with NE blank and distilled water and with niclosamide as a pharmacological control at 2 mg/L. The opercula opening and hemolymph release were the criteria for assessing mortality.

### Acetylcholinesterase inhibition assay in 96-well microplate

We use the enzyme acetylcholinesterase (AChE, E.C. 3.1.1.7 electric ell, code C3389) acquired by sigma Aldrich. A quantity of 30 mL of buffer A (Tris HCl pH: 7.8–50 mM) was used to dilute the AChE enzyme to obtain a final concentration of 66.6 U/mL. We added 1% albumin for stabilization and stored at −2°C. Additionally, we prepared buffer B: 0.067 M sodium phosphate at pH 6.85. Acetonitrile was the solvent used to prepare the 1 mM para-nitrophenyl acetate substrate. The nanoemulsion concentrations with and without the active were prepared in DMSO.

In the first step of the reaction: in the enzyme control (94 µL Buffer B, 6 µL enzyme with 1% albumin); enzyme control blank (94 µL buffer B, 6 µL tris HCl buffer with 1% albumin); to offer the same enzyme conditions, 94 µL buffer B containing inhibitor, 6 µL tris HCl buffer with 1% albumin were placed in the inhibitor blank and 94 µL buffer B containing inhibitor and 6 µL enzyme with 1% albumin at 2U in the inhibitor test/mL. Then we conditioned in the biological oxygen demand (B.O.D) at 25°C for 10 min to interact the inhibitor with the enzyme. In the second step, we completed the reaction volume with 98 µL Buffer B and 2 µL of 1 mM 4-Nitrophenyl acetate (PNPA) substrate, which was added for 20 to 20 s. After completing the reaction with a volume of 200 μL, we waited for 2 min and 30 s to start the readings on the Elisa plate reader for 20 to 20 s within a period of 5 min at a wavelength of 405 nm.

### Toxicity hemocompatibility

The toxicity of *Neomitranthes obscura* was evaluated by the hemocompatibility test, according to Bauer et al. (2012), with modifications. The compound (100 μg/mL) or saline (control) was incubated with a 13% (v/v) red blood cell suspension for 3 h at 37°C. Then, the samples were centrifuged for 3 min at 1,800 rpm, and lysis of the cells was detected by measuring hemoglobin at an absorbance of 578 nm using a microplate reader (SpectraMax, Model M4, Molecular Devices, California, United Stated). One hundred percent hemolysis (pharmacological control) was achieved by adding Triton X-100 (1%, v/v) or water to the red blood cell suspension.

### Single dose toxicity

*Neomitranthes obscura* toxicity was evaluated by the *in vivo* test, according to ANVISA. National Health Surveillance Agency (2013), with modifications. NE (1,000 mg/kg) or saline solution was injected intraperitoneally (i.p.) into the abdominal region of the mice. Then, behavior and mortality were observed for 24 h.

### *In silico* assays

The prediction of the ecotoxicity profile of the major essential oil compounds was performed by ADMET Predictor™ (version 9.5, Simulations Plus, Lancaster, CA). The terpenoids were compiled in the format of the simplified molecular-input line-entry system (SMILES) and entered into ADMET Predictor™. The endpoints analyzed were bioconcentration, biodegradation, impact at three different trophic levels (*Tetrahymena pyriformis*, water flea (*Daphnia*), and fathead minnow), and effects due to interference with sex hormones (estrogen and androgen disruptors).

### Statistical analysis

Statistical analysis was performed using the Statgraph and Prism 6 GraphPad programs (GraphPad Software) using two-way ANOVA followed by Tukey’s test with *p* < 0.001. Linear regression with *p* < 0.0001 The probit analysis was performed using Statgraphics Plus software v.5.1 (Stat Easy Co., Minneapolis, United States).

## Results

### Essential oil extraction

**FIGURE 1:**
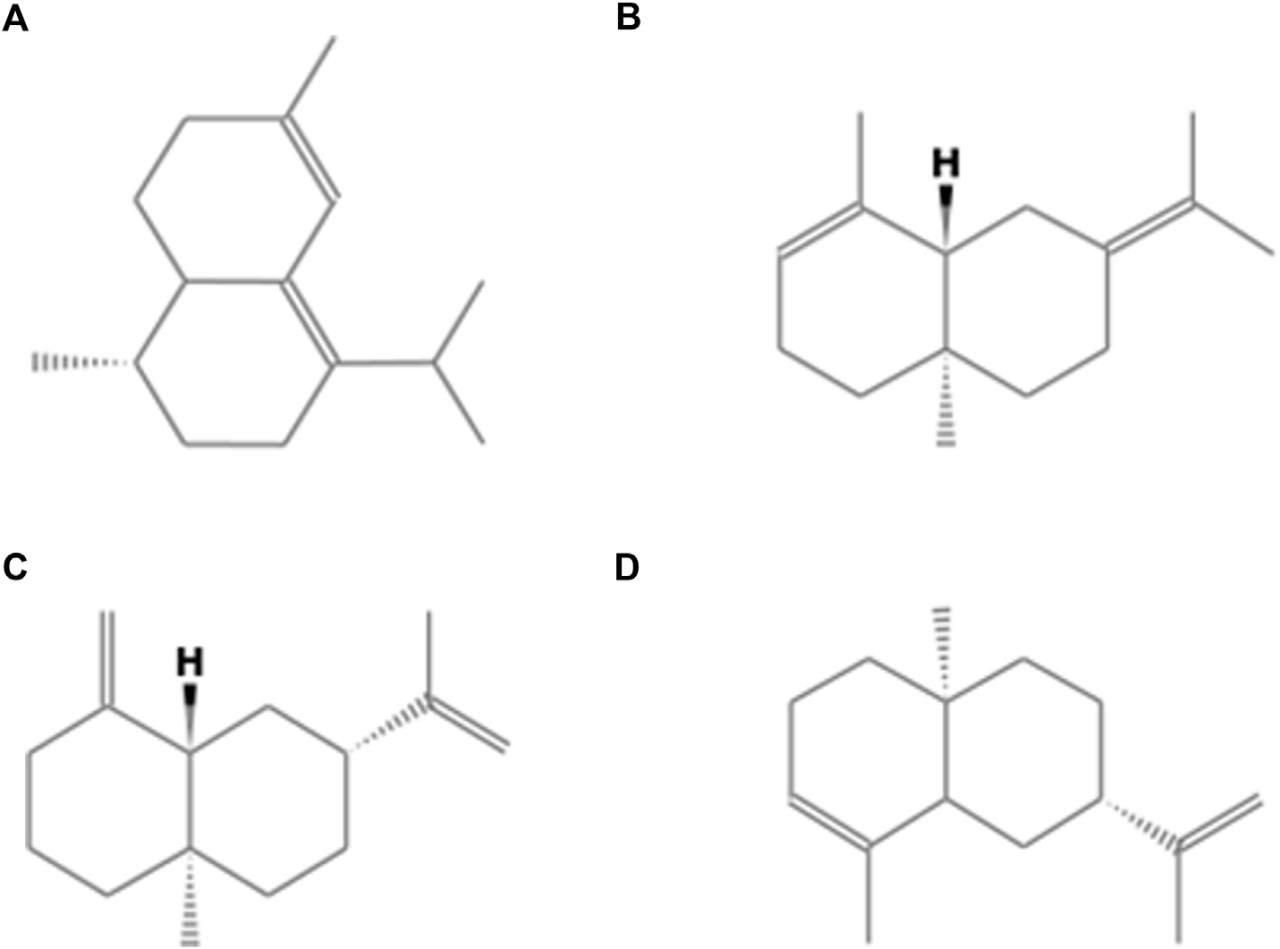
Chemical structures of major compounds from *Neomitranthes obscura* essential oil. **(A)** zonarene, **(B)** seline-3,7 (11)-diene, **(C)** β-selinene, and **(D)** α-selinene.

The *N. obscura* essential oil from fresh leaves showed a translucent appearance, light yellow color, and a yield of 1.2%. A total of 27 substances were identified, representing 90.18% of the oil (Table 1). The largest fraction was composed of sesquiterpenes (78.82%). In addition, the major compounds (Figure 1) were zonarene (22.4%), seline-3,7(11)-diene (16.2%), β-selinene (8.6%) and α-selinene (5.9%).

**TABLE 1:** Chemical characterization of *Neomitranthes obscura* essential oil from leaves obtained from GC-MS chromatogram.

	RT	IA exp	IA lit	Substância	%
1	5.570	928	932	α-Pinene	5.44
2	6.765	973	974	β-Pinene	4.51
3	7.099	985	998	Myrcene	0.31
4	8.390	1,025	1,024	Limonene	0.69
5	8.459	1,027	1,026	1,8-Cineole	0.41
6	22.351	1,369	1,374	α-Copaene	1.12
7	23.095	1,387	1,389	β-Elemene	3.73
8	24.121	1,411	1,417	β-Caryophyllene	5.40
9	24.580	1,423	1,434	γ-Elemene	2.19
10	24.798	1,428	1,437	α-Guaiene	1.17
11	24.900	1,431	1,439	Aromadendrene	0.39
12	25.578	1,447	1,452	α-Humulene	0.77
14	26.332	1,466	1,473	4,11-selinadiene	0.60
15	26.464	1,469	1,479	Amorpha-4,7(11)-diene	1.15
17	26.940	1,481	1,489	β-Selinene	8.59
19	27.233	1,498	1,498	α-Selinene	5.87
21	27.782	1,502	1,505	β-Bisabolene	2.30
23	28.131	1,511	1,520	7-epi-α-Selinene	2.18
25	28.839	1,529	1,528	Zonarene	22.38
26	29.026	1,534	1,545	Selina-3,7(11)-diene	16.18
27	29.646	1,550	1,559	Germacrene B	4.80
Total					90.18
Hydrocarbon monoterpenes					10.95
Oxygenated monoterpenes					0.41
Hydrocarbon sesquiterpenes					78.82

### Nanoemulsion preparation and characterization

The required HLB of the most promising formulation containing the essential oil of *N. obscura* (NE) was 14.5, presenting a bluish color, turbidity of 95.25%, droplet size of 164.5 nm, and polydispersion index of 0.309. The selection criterion was a smaller droplet size, followed by the PdI value. The other formulations are described in Table 2.

**TABLE 2:** Composition of formulations with *Neomitranthes obscura* essential oil.

HLB	Span 80 (%)	Tween 80 (%)	EO (%)	Água (%)	Turbidity (%)	Droplet size (nm)	PdI
12.0	1.402	3.598	5.0	90.0	97.57	322.5	0.334
12.5	1.168	3.831	5.0	90.0	98.70	204.1	0.290
13.0	0.9345	4.065	5.0	90.0	97.93	216.3	0.327
13.5	0.7005	4.299	5.0	90.0	92.77	204.5	0.318
14.0	0.467	4.532	5.0	90.0	90.80	217.7	0.344
14.5	0.2335	4.766	5.0	90.0	95.25	164.5	0.309
15.0	0.0	5.0	5.0	90.0	96.87	262.4	0.320

*EO, essential oil; HLB, hydrophilic-lipophilic balance; PdI, polidispersity index.

### Molluskicidal assay

**FIGURE 2:**
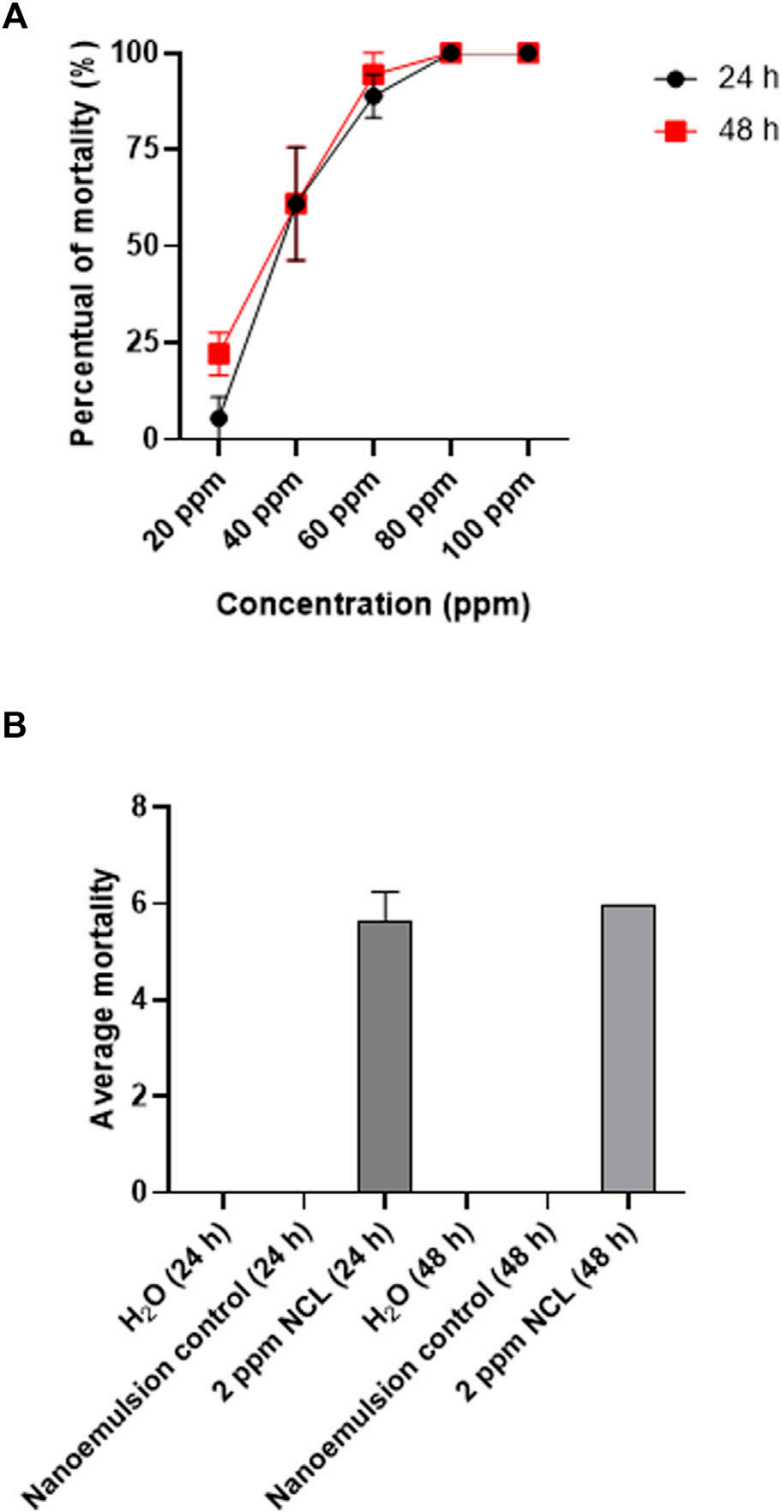
**(A)** Molluskicidal effect of the *Neomitranthes obscura* nanoemulsion on *Biomphalaria* glabrata adults exposed for 48 h. **(B)** Controls. These tests were repeated three times in three different periods (*n* = 9). R2: 0.9994, *p*-value: <0.0001.

Treatment with the essential oil nanoemulsion against adult (10–12 mm) *B. glabrata* caused a toxic effect, with LC_50_ and LC_90_ values of 39.4 (24.6–52.7) and 55.4 ppm (46.6–100.0), respectively, after 24 h. After 48 h, they reduced the LC_50_ and LC_90_ values to 34.0 ppm (12.6–47.7) and 55.4 ppm (43.2–109.5), respectively. Figure 2A shows the average mortality of adult mollusks after 48 h, showing a lethality of 100% of the population at 80 ppm in 24 h. The negative and blank controls of NE did not present mortality (Figure 2B).

**FIGURE 3:**
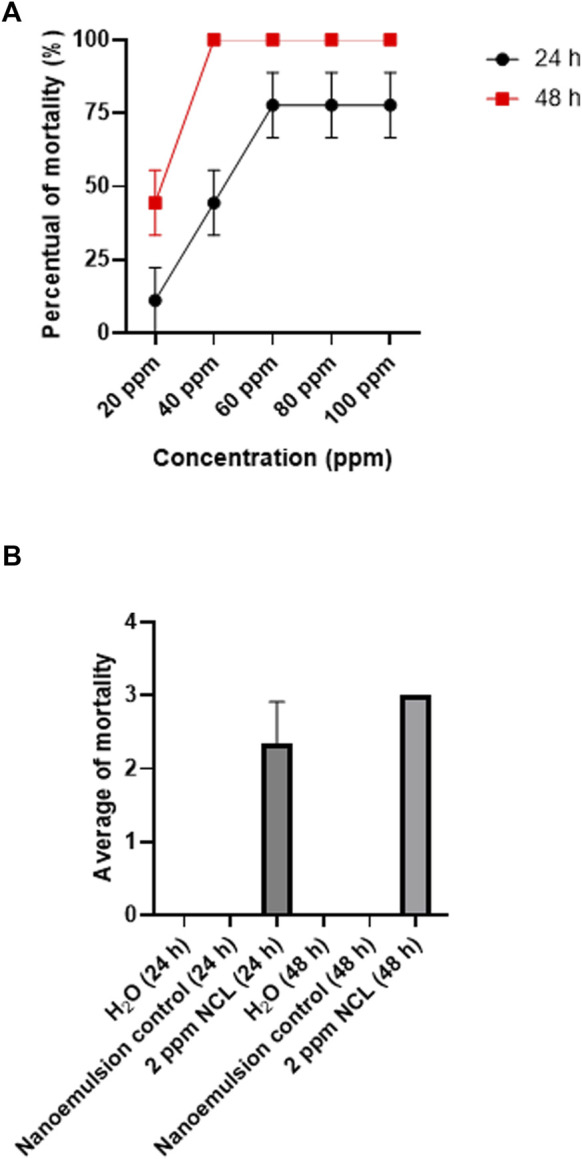
Molluskicide test on juvenile *Biomphalaria glabrata* for 48 h using NE **(A,B)**. The test was performed in triplicate on different days using 27 specimens/triplicate. These data are expressed as the mean ± S.D. Linear regression analysis, *R* ^2^: 0.8924, *p*-value: 0.0025.

The essential oil nanoemultion was also evaluated against juvenile (6–8 mm) *B. glabrata*. The LC_50_ value observed for *B. glabrata* juveniles was 20.6 ppm, and the LC_90_ was 25.05 ppm after 48 h (Figure 3A). The negative and blank controls of NE did not present mortality (Figure 3B).

### Ovicidal assay

**FIGURE 4:**
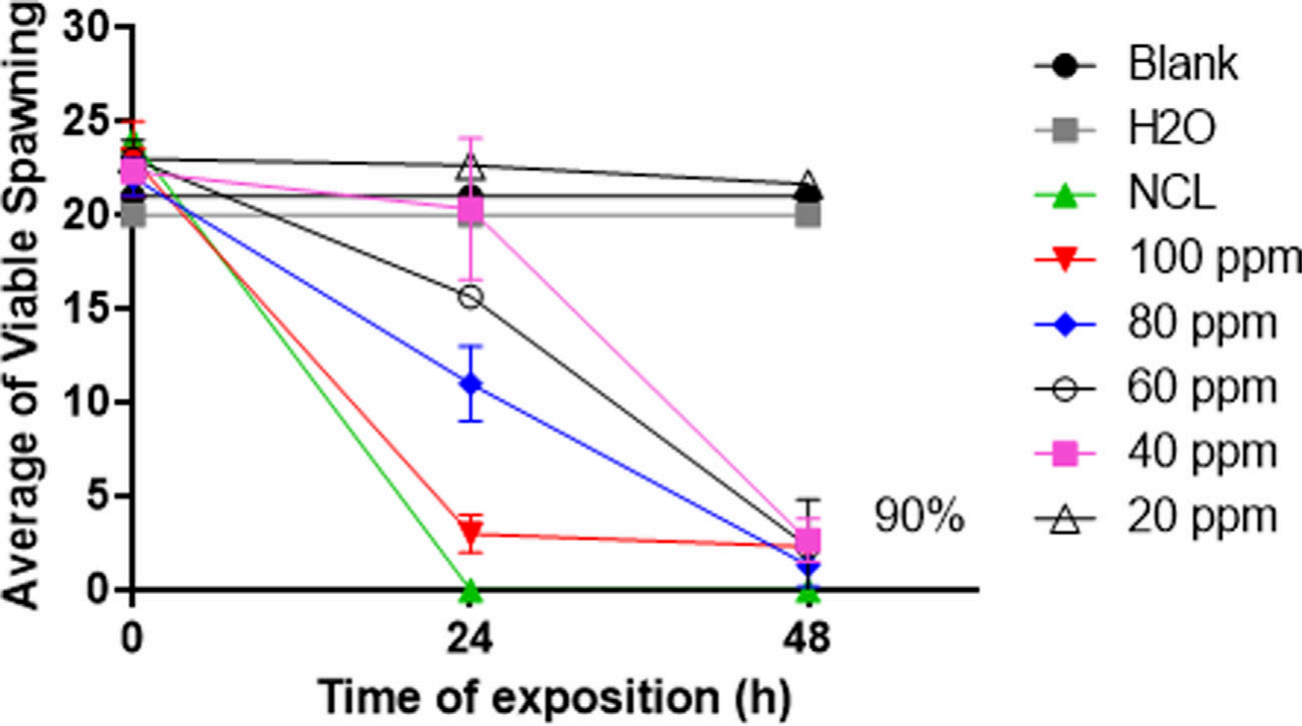
Effect of NE on the spawning of *Biomphalaria glabrata* in the period from 24 to 48 h. The test was repeated three different times in triplicate (*n* = 9). A range of 20–25 spawns was used per well. These data are expressed as the mean ± S.D. The statistical test used was ANOVA 2-way, *R* ^2^: 0.9793, *p*-value: <0.0001.

The NE effect on the spawning viability of *B. glabrata* was tested in crescent concentrations for 24 and 48 h. After 48 h, the NE at 100 ppm presented a mortality rate higher than 90% of the viable eggs (Figures 4). The lethal concentrations obtained after 48 h were an LC_50_ of 30.3 ppm and an LC_90_ of 66.25 ppm.

### Cercaricidal assay

**FIGURE 5:**
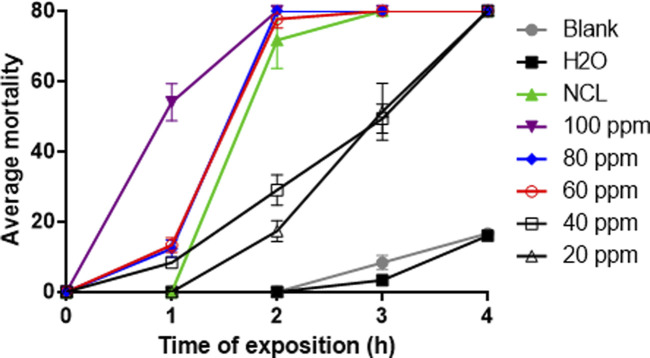
NE activity against *Schistosoma mansoni* cercariae for 4 h. The test was performed in triplicate on different days using a range of 80 cercariae per well during the testing of the samples. These data are expressed as the mean ± S.D. ANOVA 2-way, *R* ^2^: 0.9813, *p*-value: <0.0001.

The NE effect on *S. mansoni* cercariae viability was measured in crescent concentrations for up to 4 h. After 1 h of exposition, the concentration of 100 ppm NE eliminated more than 50% of cercariae, much higher than the pharmacological control. After 2 h of exposure, the concentrations of 60 ppm–100 ppm eliminated 100% of cercariae. After 4 h of exposure, all concentrations tested reduced the cercariae population by 100% (Figure 5). The lethal concentrations for the cercaricidal test are shown in Table 3.

**TABLE 3:** Lethal concentration of the *Neomitranthes obscura* nanoemulsion in cercariae after 4 h.

Hours	LC_50_ (ppm)	LC_90_ (ppm)
2	38.1	59.18
3	16.5	46.3
4	9.99	11.5

### Mechanism action of the nanoemulsion

**FIGURE 6:**
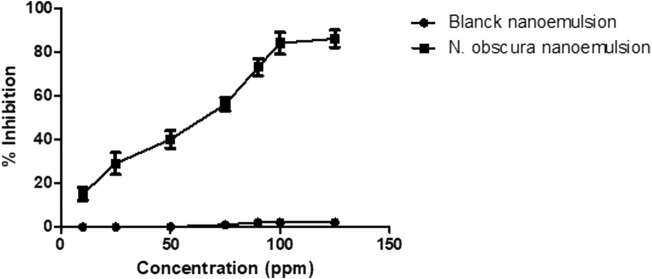
Inhibition percentage of NE against anti-cholinesterase activity. These experiments were performed in triplicate at least three distinct days.

Additionally, we investigated the nanoemulsion effect on cholinesterase enzyme activity. The nanoemulsion dose-dependently inhibited this enzyme activity (Figure 6) with maximal inhibition about 80% in the concentration of 100 ppm. In contrast, the blanck nanoemulsion did not exhibit effect on this enzyme. Therefore, the hypothetical nanoemulsion effect is through of this enzyme inhibition.

### Effects of *Neomitranthes obscura* nanoemulsion on another snail species

**FIGURE 7:**
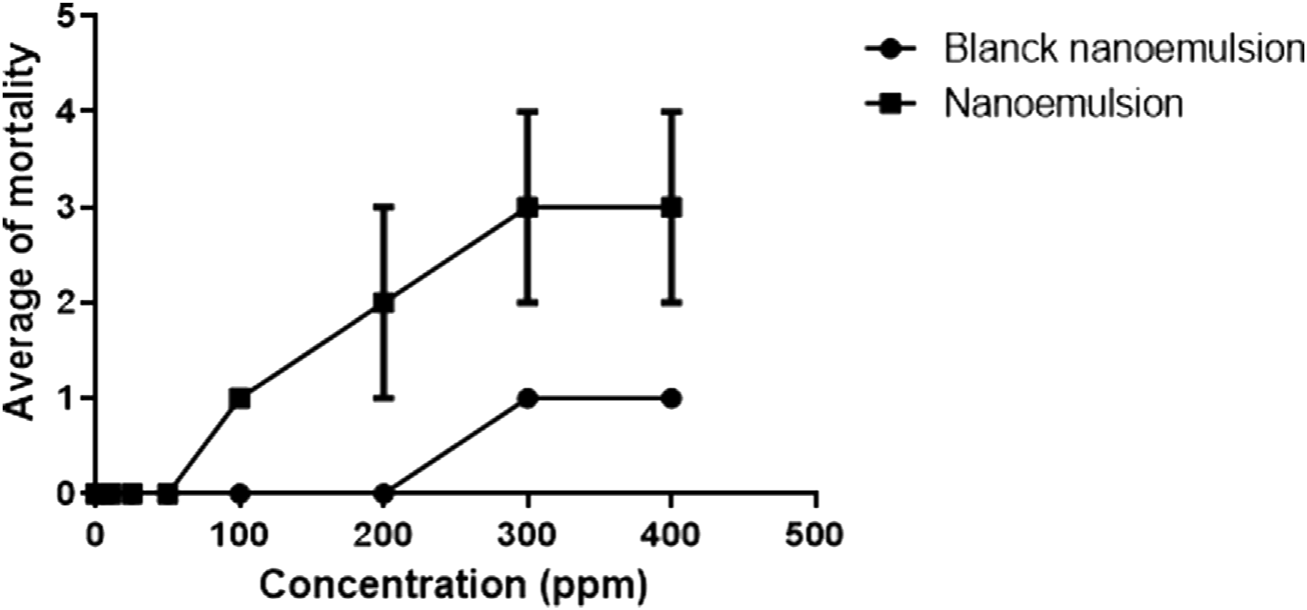
NE effect on *Mellanoides sp* viability. The snails were exposed for 48 h with NE and blanck NE. These tests were repeated three times in three different periods (*n* = 9). R2: 0.9, *p*-value: <0.0001.

The *N. obscura* nanoemulsion toxicity was evaluated on *Mellanoides sp* for 48 h caused toxicity in concentrations higher than 100 ppm with maximal toxicity in 300 ppm (Figure 7). The nanoemulsion without the active (blanck nanoemulsion) did not caused relevant mortality until 300 ppm. *In vitro* Toxicity of *Neomitranthes obscura*.

The toxicity of *N. obscura* was evaluated by an *in vitro* hemocompatibility test with red blood cells. Treatment of cells with Triton X-100 or water lysed 100% of the red blood cells (positive groups), whereas treatment with saline solution (control) resulted in no lysis. NE (100 μg/mL) lysed approximately 5% of red blood cells (data not shown), and according to Bauer et al. (2012), hemolysis below 9% means that the compound or molecule is devoid of toxicity; thus, NE can be considered a non-hemolytic or non-toxic molecule.

### Single dose toxicity

We performed an assessment of mortality resulting from the probability of survival for the 1,000 mg/kg dose (ANVISA. National Health Surveillance Agency, 2013). This demonstrated that there was no lethal or behavioral toxic effect after inoculation of *N. obscura* compounds during the 24 h of observation.

### *In silico* assay

The overall environmental toxicities of the four major chemical components of the *Neomitranthes obscura* essential oil (zonarene, selina-3,7(11)-diene, α-selineno, and β-selineno) are presented in Table 4.

**TABLE 4:** Results of the compound ecotoxicity. The endpoints legend evaluated for each compound are defined: BCF, bioconcentration factor value; BOD, biodegradation—categorize the compounds as positive (readily biodegradable) if %biodegradation is greater than or equal to 60% and as negative otherwise; Th_pyr_pIGC50, concentration of toxicant needed to inhibit 50% growth (IGC50) of *Tetrahymena pyriformis* after *approximately* 40 h of exposure; Daphnia_LC50, concentration (mg/L) of compound required to kill 50% of a *D. magna* population; Minnow_LC50, concentration (mg/L) of a compound that kills 50% of a population of minnows; Andro_Filter and Estro_Filter, assess a compound’s likelihood of binding to the androgen/estrogen receptor.

Compounds	BCF	BOD	Aquatic toxicity			Endocrine receptor binding	
			Th_pyr_pIGC50	*Daphnia*_LC50	Minnow_LC50	Andro_Filter	Estro_Filter
Zonarene	1,497.046	No	1.003	11.855	0.421	Non-toxic	Non-toxic
Selina-3,7(11)-diene	1,020.05	No	1.076	2.262	1.211	Non-toxic	Non-toxic
α-selineno	1,943.41	No	1.089	1.629	0.142	Toxic	Non-toxic
β-selineno	1,951.705	No	1.287	2.110	0.369	Toxic	Non-toxic
Niclosamide	6.65	No	1.968	1.752	3.612	Toxic	Non-toxic

The *in silico* data indicate some potential environmental toxicity for the major essential oil compounds, especially the bioccumulative potential and biodegradability. On the other hand, the two essential oil compounds with the highest percentage (zonarene and selina-3,7(11)-diene) are not predicted to disrupt the endocrine receptors.

## Discussion

Currently, schistosomiasis control includes methods such as prophylaxis, mollusk control, and the use of antiparasitics in the treatment of the disease (Neglected_diseases, 2022). The control of intermediate hosts of parasites of the *Schistosoma* genus, such as *Biomphalaria glabrata*, is widely used, but it is dependent on the few registered molluskicidal agents commercially available that have limitations, such as the occurrence of biological resistance to these chemical pesticides. In addition, they present high environmental residual effects, generating a negative impact on the local ecosystem (Colley et al., 2014; Neglected_diseases, 2022). Therefore, studies involving the search and development of new drugs with molluskicidal and/or antiparasitic potential against *Schistosoma* are necessary. Plants are a good source of active metabolites that can generate bioproducts with effectiveness, economic viability, and low environmental impact (Ramos et al., 2010; Amaral et al., 2014; Araújo et al., 2019).

Plants of the Myrtaceae family are described in the literature despite their pharmacological and biological activities, such as insecticidal, larvicidal, and anticholinesterase activities (Ramos et al., 2010).

The essential oil extraction showed a satisfactory yield of 1.2%, and the composition of the essential oil (EO) from leaves of *N. obscura* was previously described with 87% and 82.7% of sesquiterpene fractions. The major components were α-cadinene (16.2%), seline-3.7(11)-diene (10.6%), cis-nerolidol (19.3%), trans-nerolidol (17.1%), and β-bisabolene (11.7%) (Liu et al., 2014; Sobhani et al., 2018). Other studies compared the EO of *N. obscura* in leaves and fruits and described chemical profiles with selin-3.7(11)-diene, trans-dauca-4(11)7-diene, β-caryophyllene, germacrene B, α, and β selinenes. These results reinforce the findings in our work with the presence of seline-3,7-(11)-diene, selinene fractions, β-caryophyllene, and germacrene B (Amaral et al., 2013; Amaral et al., 2014).

In our work, the chemical EO from leaves of *N. obscura* showed the highest fraction of sesquiterpenes, corresponding to 78.8%, thus corroborating the highest fractions described by other authors 25–28). The major terpenoid was zonarene (22%), followed by selina-3.7(11)-diene (16.2%). The metabolites β-selinene, α-selinene, β-caryophyllene, and germacrene B also appeared in the *N. obscura* EO analyzed, corroborating the results of the aforementioned authors. The constituent zonarene, a compound that until then had not been described as a chemical component of *N. obscura* oil, may play a key role in molluskicidal activity since it is present in greater quantities. The production of this skeleton-type of sesquiterpene is common in the biosynthetic pathway in Myrtaceae. Likewise, minor substances such as the monoterpenes α-pinene and β-pinene are possibly relevant to the chemical profile and bioactivity of the species. Variations between products of metabolism among individuals of the same species are common due to different environmental pressures, which result in different metabolic expression pathways (Amaral et al., 2013; Amaral et al., 2014).

Chemical analysis of the essential oil of *N. obscura* allowed the identification of 90.18% of its components. Of these, 78.82% were shown to be hydrocarbon sesquiterpenes, 10.95% hydrocarbon monoterpenes and only 0.41% oxygenated monoterpenes. The 4 major components of the essential oil are all hydrocarbon sesquiterpenes: zonarene (22.38%), selin-3,7(11)-diene (16.18%), beta-selinene (8.59%) and alpha-selinene (5.87%). These four majority substances together add up to more than half of the essential oil components (53.02%). Therefore, we could affirm that the sesquiterpene hydrocarbons are responsible, at least in part, for the activity presented by the essential oil of *N. obscura*".

The nanoemulsion is a product of operational simplicity, is effective, has a low cost of production, and allows the dispersion of lipophilic agents, such as essential oils, in aquatic environments, presenting itself ideally for the control of mollusks of medical interest. The extremely low droplet size provides greater resistance to the effects of cremation and sedimentation, suggesting good physicochemical stability (Victório et al., 2018; Araújo et al., 2019; Feng et al., 2021).

The PIT method (Mustafa and Hussein, 2020; Feng et al., 2021) was used because it forms low interfacial tension between the non-ionic surfactants and the oil phase, favoring emulsification together with a reduced drip velocity of the aqueous phase and constant homogenization by magnetic stirring. In addition, this method does not use organic solvents and allows reproducibility on an industrial scale (El-Ekiaby, 2019; Mustafa and Hussein, 2020; Feng et al., 2021; Marhamati et al., 2021). The most promising formulation with *N. obscura* essential oil presented a proportion of surfactants of 0.2335% for Span 80, 4.766% for Tween 80, 5% for essential oil, and 90% for distilled water. The required HLB of 14.5 suggests that the essential oil has some hydrophilic characteristics. Additionally, the HLB 14.5 formulation showed a translucent bluish color characteristic of light scattering in colloidal systems with reduced particle size defined by the Tyndall effect (El-Ekiaby, 2019; Marhamati et al., 2021). In addition, the turbidity evaluates the transparency of the system; values close to 100% indicate a lower scattering and absorption of light. All formulations in table 2 presented values that indicate the presence of reduced-size droplets. For a dispersion to be classified as a nanoemulsion, it must have a droplet size of 20–200 nm and PdI values below 0.3 to be considered a monodisperse system (El-Ekiaby, 2019; Matos et al., 2020). Within this context, the most promising formulation fits as a nanoemulsion with a particle size of 164.5 nm and 0.309 PdI.

NE showed molluskicidal activity with an LC_50_ of 35.3 ppm and an LC_90_ of 53.9 ppm after 48 h. The WHO classifies a plant extract with molluskicidal properties if the LC_90_ value is below 100 ppm after 48 h (OMS-Organização Mundial da saúde, 1983). In this way, NE can be classified as a promising agent for the control of *B. glabrata*, and since the nanoemulsion is stable, operationally simple and low cost, industrial production is possible, making it an alternative for use in large portions of water.

The NE estimated for 24 h showed a reduction of 80% in *B. glabrata* egg viability at 100 ppm, and at 48 h, it was reduced by more than 90% at 60 ppm. Watanabe (Watanabe, 1997) reported the great importance of tests on embryonic stages in *Biomphalaria* sp. serving as bioindicators in polluted waters and tests performed as biomarkers in tests and mutagenicity.

In the cercaricidal assay, NE presented a mortality of more than 90% of the cercariae in the second hour at a concentration of 60 ppm, and after 4 h, even the lowest concentration of 20 ppm eliminated all cercariae (LC_90_ = 11.5 ppm), proving to be effective when compared to the control groups (*p*-value: <0.0001). The data obtained in this work showed experimental similarity with the study published by Albagouri et al. (2014), who demonstrated the cercaricidal activity of different Sudanese plants, such as the LC_50_ and LC_90_ found in this study, with *N. obscura* presenting values below 20 ppm.

Organophosphate pesticides and carbamates cause toxicity acting on AChE (Garate et al., 2020). Additionally, niclosamide possess a similar effect on molluscks (Wang et al., 2016). Therefore, we evaluated the NE effect on AChE activity and observed NE inhibiting the enzyme activity. Curiously, the main essential oil constituent and α-selinene did not possess description of activity on this enzyme. However, seline-3,7(11)-diene was predicted to interact with AChE and selina-3,7(11)-diene (Menghini et al., 2021), β-selinene showed strongest AChE inhibition (Liu et al., 2021).

Niclosamide treatment cause ambiental toxicity (Leaka et al., 2020). Interestingly, *N. obscura* nanoemulsion was not toxic for *Mellanoides sp*. Therefore, this nanoemulsion would be a satisfactory alternative for *B. glabrata* control.

*N. obscura* hemolysis below 10% means that the compound or molecule is devoid of toxicity and, therefore, NE is within the allowed limit of 5% and can be considered non-hemolytic or non-toxic molecules. In addition, the toxicity assessment was performed *in vivo*, observing the probability of mortality at a dose of 1,000 mg/kg. *N. obscura* did not cause lethal or induced toxic behavioral effects in mice during 24 h of observation. Regarding the *in silico* environmental toxicity assay, the major compounds of the *Neomitranthes obscura* essential oil (zonarene, selina-3,7(11)-diene, α-selineno, and β-selineno) are predicted to have a higher bioaccumulative potential than niclosamide. Nevertheless, none of them presented an alarming bioaccumulative risk. A substance is considered bioaccumulative for REACH if the BCF value is greater than 2,000 (Lombardo et al., 2010).

All the compounds tested are predicted not to be biodegradable. Therefore, attention must be paid to the amount of waste generated in the environment and its potential harm. In this way, we emphasize that the nanoemulsion presents very low amounts of the bioaccumulative compounds mentioned above, and even so, they are compounds with low environmental impact, being ideal for use in lakes, ponds, dams, streams. However, we need to perform environmental toxicity tests and quantify the level of nanoformulation by-product. After we get this information, we can have a clearer notion of how and in which situation we can use the nanoformulation.

From the analysis of the impact at three different trophic levels, the four major compounds presented similar potential to inhibit *T. pyriformis* growth than niclosamide. In the *D. magna* model, the 20% most toxic compounds presented an LC_50_ less than approximately 3.0. Therefore, only the major compound zonarene presented a small toxicity prediction; indeed, niclosamide is predicted to be more toxic than zonarene for *D. magna*. The *Pimephales promelas* model showed that approximately 20% of the most toxic compounds presented an IGC_50_ less than 2.0. Therefore, the compounds selina-3,7(11)-diene, α-selineno, and β-selineno presented potential toxicity. Zonarene is predicted to be 9 times more toxic to *P. promela* than niclosamide.

Regarding the estrogen receptor, most of the tested compounds are predicted to have a non-toxic effect. On the other hand, α-selineno and β-selineno exhibited the potential to interact with the androgen receptor. Therefore, these compounds present a risk of adverse health outcomes, including cancer, reproductive impairment, cognitive deficits, and obesity.

The endocrine toxicological results indicated that zonarene presented a low potential to interact with the tested receptors. This result suggests a low endocrine toxicological effect of this compound compared to that of niclosamide. Indeed, some recent works report the inhibition of androgen receptors by niclosamide (Liu et al., 2014; Sobhani et al., 2018; Parikh et al., 2021).

Thus, the present study demonstrated the molluskicidal activity of the nanoemulsified essential oil from the leaves of *N. obscura* in embryos, adult, and juvenile forms of *B. glabrata* mollusks, as well as the cercaricidal activity. Consequently, the biotechnological product developed from *N. obscura* exhibited action at different stages of the schistosomiasis transmission cycle, thus being a promising alternative in the control of this disease.

## Conclusion

This work demonstrated the potential of the nanoemulsion of the essential oil of *Neomitranthes obscura* as molluscicide agent against the host species *Biomphalaria glabrata* and the human infective form cercariae.

## Data Availability

The raw data supporting the conclusion of this article will be made available by the authors, without undue reservation.

## Abbreviations

AChE, Acetylcholinesterase; AI, Arithmetic index; Andro_Filter and Estro_Filter, Assess a compound’s likelihood of binding to the androgen/estrogen receptor; BCF, Bioconcentration factor value; BOD, Biodegradation; B.O.D, Biological Oxygen Demand; *Daphnia*_LC50, Concentration (mg/L) of compound required to kill 50% of a D. magna population; DLS, Dynamic light scattering; EO, Essential oil; FID, Ionization detector; GC, Gas chromatographic; HCl, Hydrochloric acid; HLB, Hydrophilic-lipophilic balance; LC, Lethal concentration; Minnow_LC50, Concentration (mg/L) of a compound that kills 50% of a population of minnows; MS, Mass spectrometry; NEs, Nanoemulsions; O/W, Oil-in-water; PNPA, 4-Nitrophenyl acetate; PdI, Polydispersity index; PIT, Phase inversion in temperature; SMILES, Simplified molecular-input line-entry system; Th_pyr_pIGC50, Concentration of toxicant needed to inhibit 50% growth (IGC50) of *Tetrahymena* pyriformis after approximately 40 h of exposure; v/v, Volume/volume; w/w, Water-in-water; WHO, World Health Organization.
